# ChatGPT Versus Consultants: Blinded Evaluation on Answering Otorhinolaryngology Case–Based Questions

**DOI:** 10.2196/49183

**Published:** 2023-12-05

**Authors:** Christoph Raphael Buhr, Harry Smith, Tilman Huppertz, Katharina Bahr-Hamm, Christoph Matthias, Andrew Blaikie, Tom Kelsey, Sebastian Kuhn, Jonas Eckrich

**Affiliations:** 1 Department of Otorhinolaryngology University Medical Center of the Johannes Gutenberg-University Mainz Mainz Germany; 2 School of Medicine University of St Andrews St Andrews United Kingdom; 3 School of Computer Science University of St Andrews St Andrews United Kingdom; 4 Institute of Digital Medicine Philipps-University Marburg and University Hospital of Giessen and Marburg Marburg Germany

**Keywords:** large language models, LLMs, LLM, artificial intelligence, AI, ChatGPT, otorhinolaryngology, ORL, digital health, chatbots, global health, low- and middle-income countries, telemedicine, telehealth, language model, chatbot

## Abstract

**Background:**

Large language models (LLMs), such as ChatGPT (Open AI), are increasingly used in medicine and supplement standard search engines as information sources. This leads to more “consultations” of LLMs about personal medical symptoms.

**Objective:**

This study aims to evaluate ChatGPT’s performance in answering clinical case–based questions in otorhinolaryngology (ORL) in comparison to ORL consultants’ answers.

**Methods:**

We used 41 case-based questions from established ORL study books and past German state examinations for doctors. The questions were answered by both ORL consultants and ChatGPT 3. ORL consultants rated all responses, except their own, on medical adequacy, conciseness, coherence, and comprehensibility using a 6-point Likert scale. They also identified (in a blinded setting) if the answer was created by an ORL consultant or ChatGPT. Additionally, the character count was compared. Due to the rapidly evolving pace of technology, a comparison between responses generated by ChatGPT 3 and ChatGPT 4 was included to give an insight into the evolving potential of LLMs.

**Results:**

Ratings in all categories were significantly higher for ORL consultants (*P*<.001). Although inferior to the scores of the ORL consultants, ChatGPT’s scores were relatively higher in semantic categories (conciseness, coherence, and comprehensibility) compared to medical adequacy. ORL consultants identified ChatGPT as the source correctly in 98.4% (121/123) of cases. ChatGPT’s answers had a significantly higher character count compared to ORL consultants (*P*<.001). Comparison between responses generated by ChatGPT 3 and ChatGPT 4 showed a slight improvement in medical accuracy as well as a better coherence of the answers provided. Contrarily, neither the conciseness (*P*=.06) nor the comprehensibility (*P*=.08) improved significantly despite the significant increase in the mean amount of characters by 52.5% (n= (1470-964)/964; *P*<.001).

**Conclusions:**

While ChatGPT provided longer answers to medical problems, medical adequacy and conciseness were significantly lower compared to ORL consultants’ answers. LLMs have potential as augmentative tools for medical care, but their “consultation” for medical problems carries a high risk of misinformation as their high semantic quality may mask contextual deficits.

## Introduction

The use of large language models (LLMs) is becoming increasingly common. Open access services such as Bard, Bing, and ChatGPT (Open AI) [1] have proven to be useful for a multitude of everyday applications [2,3]. Some experts argue that LLM services will soon augment, supplement, or replace today’s search engines, and their application will become more common in specific areas across a broad range of established software applications [4]. The growing relevance and interest in this technology are also evident by the recent acquisitions of various artificial intelligence (AI)–specialized companies by major software corporations [5-9].

Launched in November 2022, ChatGPT has become one of the most popular LLMs. It uses a so-called “deep neural network architecture” to analyze and generate human-like language responses based on the input it receives. “Deep neural network architecture” refers to a specific type of machine learning model designed to recognize patterns and relationships in data using multiple (hidden) layers of interconnected nodes or “neurons.” These nodes are organized into multiple layers, with each layer performing a specific computation on the input data and passing the results to the next layer. The term “deep” indicates the use of multiple hidden layers, allowing the detection of more complex patterns and relationships in the data compared to a “shallow” network with only 1 or 2 layers. The architecture is also classified as “neural” due to its interconnections and communication structure that are inspired by the interconnections of the human brain.

The architecture of ChatGPT is based on a transformer model, enabling it to process and understand sequences of text and generate natural language responses. A transformer model is a type of digital neural network architecture designed for natural language processing tasks, such as language translation, question answering, and text summarization. Introduced by Vaswani et al [10] in 2017, it has since become one of the most widely used architectures in natural language processing. The transformer model uses a self-attention mechanism, allowing it to capture long-range dependencies between words in a sentence without requiring sequential processing. This makes transformer models more efficient than traditional recurrent neural network architectures, which process input sequentially and are, therefore, slower and more computationally expensive. Moreover, self-attention appears to be a more interpretable class of models, linking the semantic and syntactic structure of inputs [10].

ChatGPT 3 has been trained on a vast and diverse corpus of text data, including a data set of web pages and internet content, and the BooksCorpus, a data set comprising over 11,000 books in various genres. During the training process, the model was trained to identify patterns in language, understand syntax and grammar, and generate coherent and meaningful responses to a wide variety of input prompts in different languages.

The digital age, particularly the advent of powerful search engines, has led to increasing accessibility of medical information for lay people. Thus, consulting “Dr Google” is now a common means for patients to understand their symptoms and decide how to manage their medical issues [11-15]. Considering the extensive source database and the natural language of the answers provided, LLMs will likely become a relevant “go-to” tool for initial medical consulting in the future. However, using chatbots such as ChatGPT for medical consultation is not without risk [12,16]. While search engines and LLM-based chatbots typically warn users that their generated answers do not substitute for a consultation with a specialist, many patients may trust the information and make their own diagnostic or therapeutic conclusions. Consequently, misinterpretation of their symptoms may lead to incorrect conclusions, resulting in false illness convictions, increased anxiety, and potentially dangerous self-treatment or nontreatment [17,18].

In addition, patients may harbor preconceptions that lead to conflict with their doctor, compromising the doctor-patient relationship [19,20]. The abilities and performance of LLMs, however, should not be underestimated. The fact that ChatGPT has been used as a tool to pass the United States Medical Licensing Examination (USMLE) demonstrates that LLMs can accurately answer medical questions [21,22]. Therefore, especially during times of specialist doctor shortages, long distances, and increased waiting periods, the availability of LLMs may further lower patients’ threshold to consult an LLM-based chatbot such as ChatGPT rather than a trained professional. On the other hand, access to better medical information can also be considered beneficial for understanding specific symptoms, diagnoses, or treatments. However, unsupervised medical consultation of LLMs carries a high risk of misinformation without the guidance of an experienced specialist.

The ability of LLMs to pass a general medical examination has been proven, but the performance of LLM-derived answers to specific clinical case–based questions based on symptoms and clinical cases in otorhinolaryngology (ORL) has not yet been evaluated. Given these recent developments, this pilot study aims to assess the performance of ChatGPT when answering clinical case–based questions. ORL is one of the clinical disciplines with the highest consultation rate and encompasses a wide spectrum of conditions, ranging from relatively harmless to severe and potentially life-threatening diseases. Therefore, we analyzed the performance of ChatGPT in the field of ORL and compared it to the answers of ORL consultants.

## Methods

### Study Design

**Figure 1:**
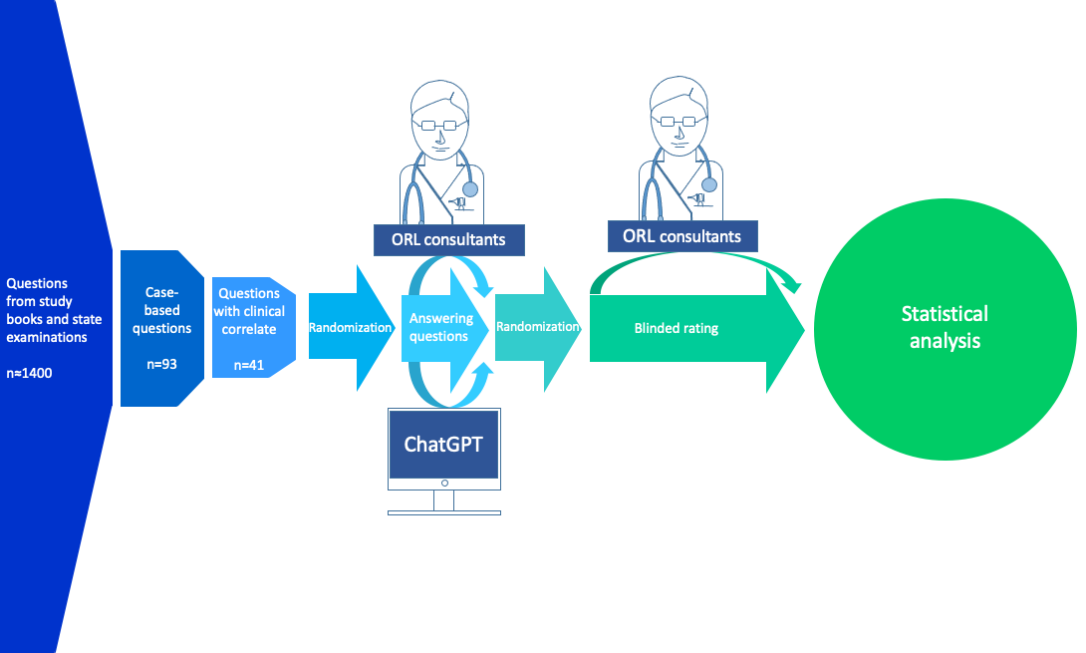
Workflow of the study. ORL: otorhinolaryngology.

The workflow of this study is shown in Figure 1. We studied established ORL textbooks and questions from previous German state examinations for doctors for case-based questions resembling realistic and authentic clinical scenarios [23,24]. Subsequently, clinical authenticity was verified by matching equivalent cases in the University Medical Center of Mainz. If cases did not have a homologous clinical correlation, they were exempt from the questionnaire. For an exemplary question, see Example S1 in Multimedia Appendix 1.

Answers to the 41 questions were recorded from 3 ORL consultants (coauthors of this paper) and the OpenAI chatbot ChatGPT 3 on March 11, 2023 [1]. Each ORL consultant (with at least 5 years of ORL-specific training) received the blinded answers of the other ORL consultants and those created by the ChatGPT LLM and was asked to rate them using a Likert scale (1=very poor and 6=excellent) for medical adequacy, conciseness, coherence, and comprehensibility. A 6-point Likert scale was chosen in order to avoid raters from taking the comfortable “neutral” position in the middle of the scale.

They then recorded whether they thought the answers were created by an ORL consultant or the ChatGPT LLM. After normality testing of the ratings using D’Agostino and Pearson test, the character count for every answer was recorded and compared using the Mann-Whitney *U* test with Prism for Windows (version 9.5.1; GraphPad Software).

Since this study aimed for maintaining a low barrier setup, simulating widespread availability, and considering a global health perspective, the experimental setup deliberately opted for the freely available versions of ChatGPT. However, challenged by the rapidly evolving pace of technology, a comparison between responses generated by ChatGPT 3 and ChatGPT 4 was included to give an insight into the evolving potential of LLMs. Ratings for answers provided by ChatGPT 3 and ChatGPT 4 were compared using the Mann-Whitney *U* test. As the amount of characters showed a Gaussian distribution, the 2-tailed *t* test was used.

### Ethical Considerations

Written correspondence of March 3, 2023, with the ethics committee of the regional medical association Rhineland-Palatinate determined that there is no need for any specific ethics approval due to the use of anonymous text-based questions.

## Results

Cumulative results of ratings in every category were significantly higher for the answers given by the ORL consultants in comparison to the ChatGPT LLM (*P*<.001), with a similar range of ratings for medical adequacy and coherence and a broader range for conciseness and comprehensibility.

In detail, medical adequacy was rated with a median of 6 (IQR 5-6; range 1-6) for the ORL consultants compared to a 4 (IQR 4-5; range 1-6) for ChatGPT LLM (*P*<.001) when tested with the Mann-Whitney *U* test. Conciseness was rated with a median 6 (IQR 6-6; range 4-6) for the ORL consultants compared to a 4 (IQR 3-5; range 2-6) for ChatGPT LLM (*P*<.001) when tested with the Mann-Whitney *U* test. Furthermore, coherence was rated with a median of 6 (IQR 5-6; range 2-6) for the ORL consultants compared to a 5 (IQR 4-5; range 2-6) for ChatGPT LLM (*P*<.001) when tested with the Mann-Whitney *U* test, and comprehensibility was rated with a median of 6 (IQR 6-6; range 4-6) for the ORL consultants and 6 (IQR 5-6; range 2-6) for ChatGPT LLM (*P*<.001) when tested with the Mann-Whitney *U* test.

**Figure 2:**
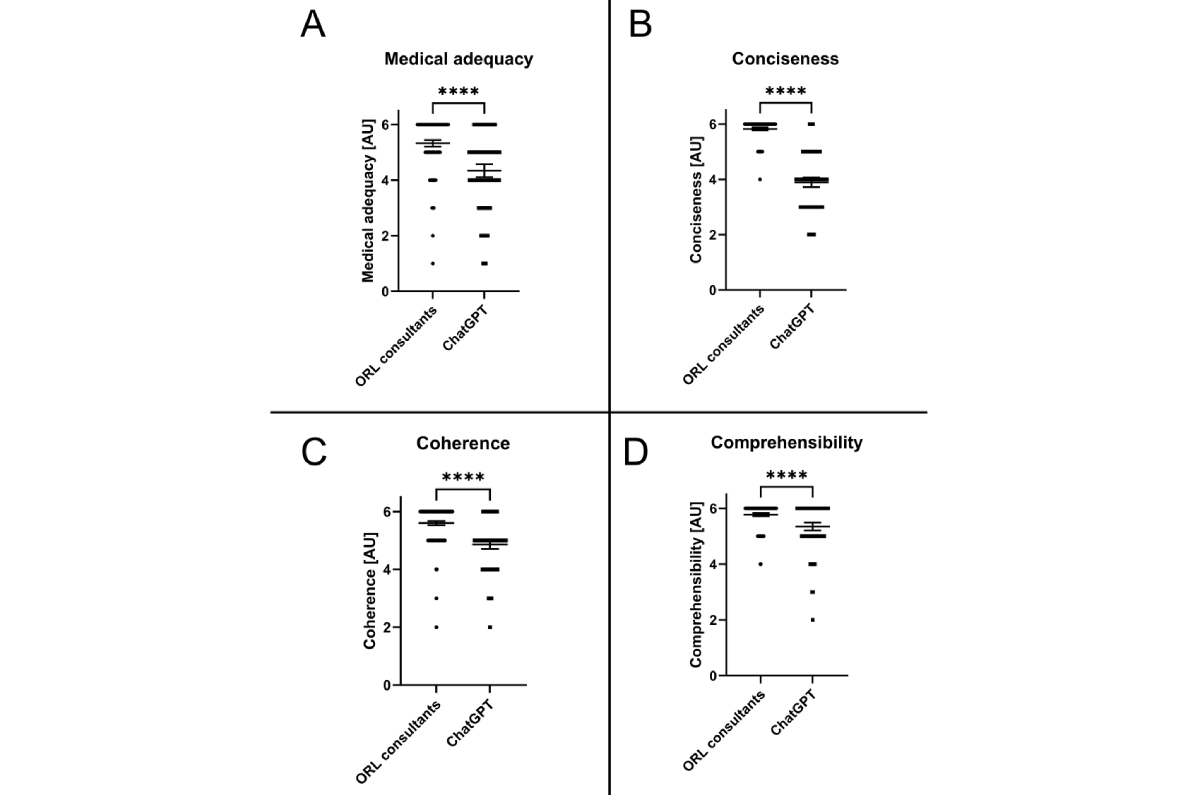
Comparison between ORL consultants and the LLM (ChatGPT) for all evaluated categories. Data shown as a scatter dot blot with each point resembling an absolute value (bar width resembling a high amount of individual values). Horizontal lines represent mean (95% CI). The nonparametric Mann-Whitney U test was used to compare the 2 groups. Cumulative results of ratings for (A) medical adequacy, (B) conciseness, (C) coherence, and (D) comprehensibility. ORL: otorhinolaryngology. ****P<.001.

Comparative results of statistical testing are shown in Table 1 and Figure 2. Scores for all 3 ORL consultants were combined and compared to the ratings of answers by the ChatGPT LLM. Individual ratings of all ORL consultants in comparison to ratings for the answers provided by the ChatGPT LLM are shown in Figures S1-S5 in Multimedia Appendix 1.

**Table 1:** Comparative results^a^.

Result and source		Ratings, n		Values, mean (SD)		Rating, median (IQR)		Rating, 95% CI
**Medical adequacy**								
	ORL^b^ consultants	246	5.3 (0.9)		6 (5-6)		5-6	
	LLM^c^ (ChatGPT)	123	4.3 (1.3)		4 (4-5)		4-5	
**Conciseness**								
	ORL consultants	246	5.8 (0.4)		6 (6-6)		6-6	
	LLM (ChatGPT)	123	3.9 (1.0)		4 (3-5)		4-4	
**Coherence**								
	ORL consultants	246	5.6 (0.6)		6 (5-6)		6-6	
	LLM (ChatGPT)	123	4.9 (0.8)		5 (4-5)		5-5	
**Comprehensibility**								
	ORL consultants	246	5.8 (0.5)		6 (6-6)		6-6	
	LLM (ChatGPT)	123	5.4 (0.8)		6 (5-6)		5-6	

^a^*P*<.001 when tested with the Mann-Whitney *U* test.

^b^ORL: otorhinolaryngology.

^c^LLM: large language model.

**Figure 3:**
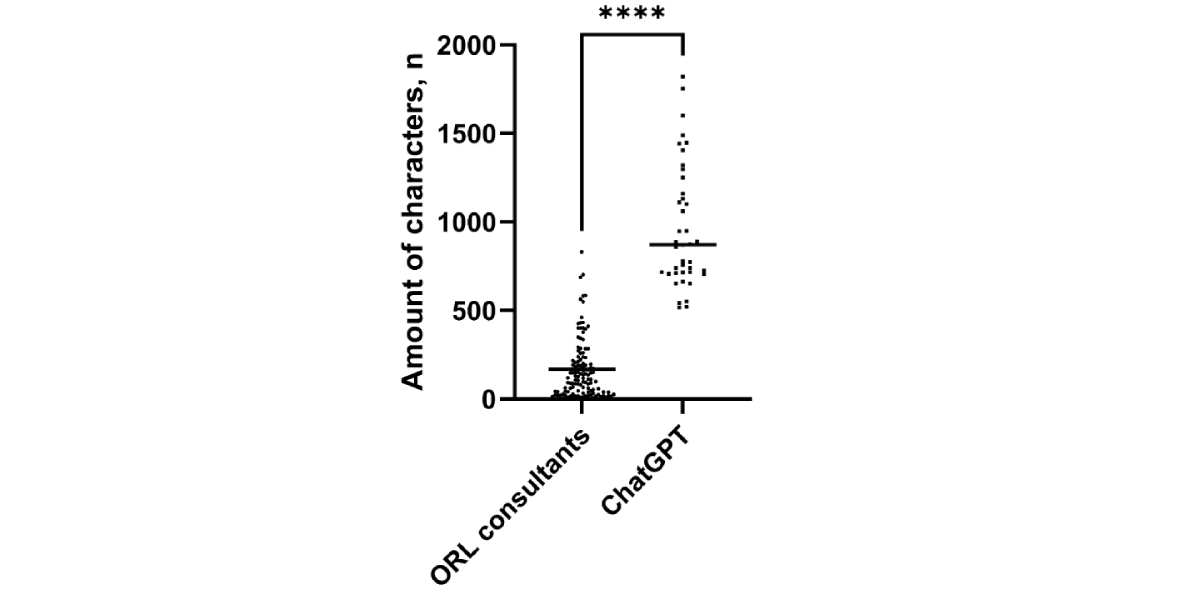
The number of characters per answer used by ORL consultants and ChatGPT. Data shown as a scatter dot blot with each point resembling an absolute value. Horizontal lines represent the median. The nonparametric Mann-Whitney U test was used to compare the 2 groups. ORL: otorhinolaryngology. ****P<.001.

The amount of characters of answers provided by the ORL consultants was significantly lower (median 119.0 (range 4-831; IQR 38.0-223.0) compared to (median 870.0 (IQR 712.5-1205.0) characters per answer for answers by ChatGPT LLM (*P*<.001) when tested with the Mann-Whitney *U* test (Figure 3). For 98.4% (369/375) of the answers, the ORL consultants correctly identified the source of the answer.

The supplemented comparison between responses generated by ChatGPT 3 and ChatGPT 4 showed a slight improvement in medical accuracy (*P*=.03). Additionally, ChatGPT 4 was rated with a better coherence of the answers provided (*P*=.005).

**Figure 4:**
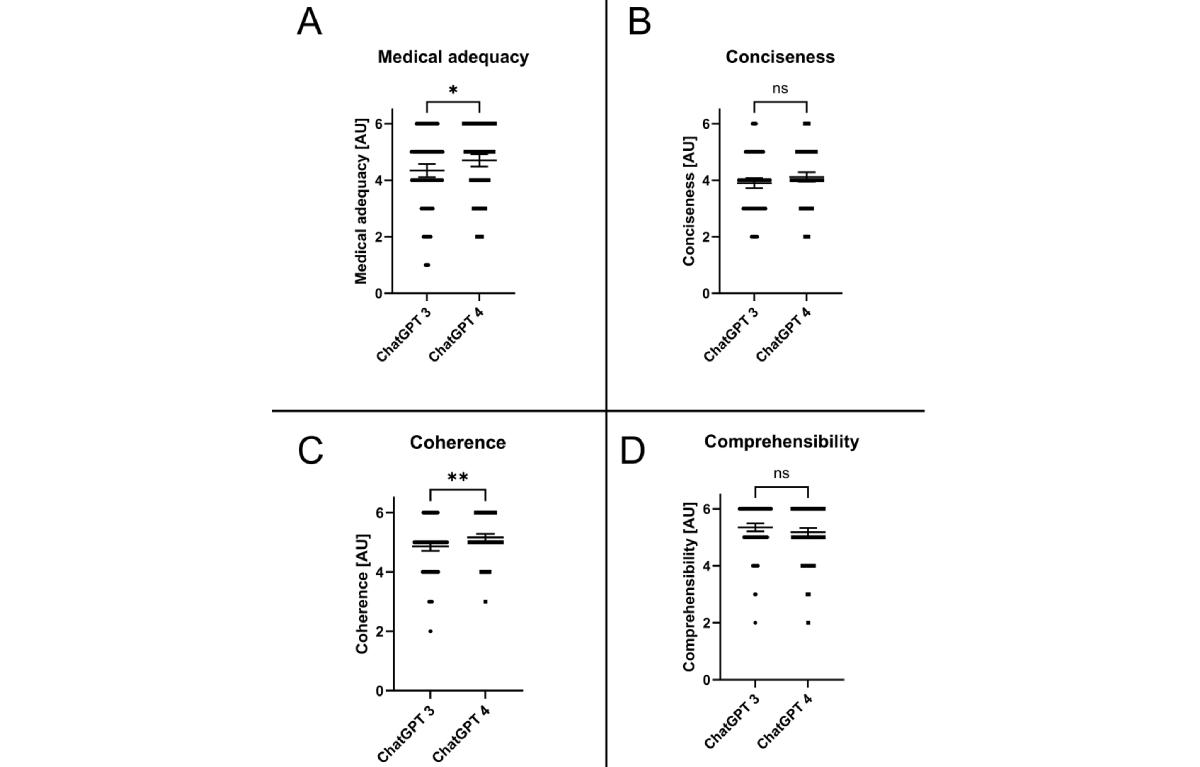
Comparison between LLMs (ChatGPT 3 vs ChatGPT4) for all evaluated categories. Data shown as a scatter dot blot with each point resembling an absolute value (bar width resembling a high amount of individual values). Horizontal lines represent mean (95% CI). The nonparametric Mann-Whitney U test was used to compare the 2 groups. Cumulative results of ratings for (A) medical adequacy, (B) conciseness, (C) coherence, and (D) comprehensibility. ns: not significantly different. *P<.05; **P<.01.

**Figure 5:**
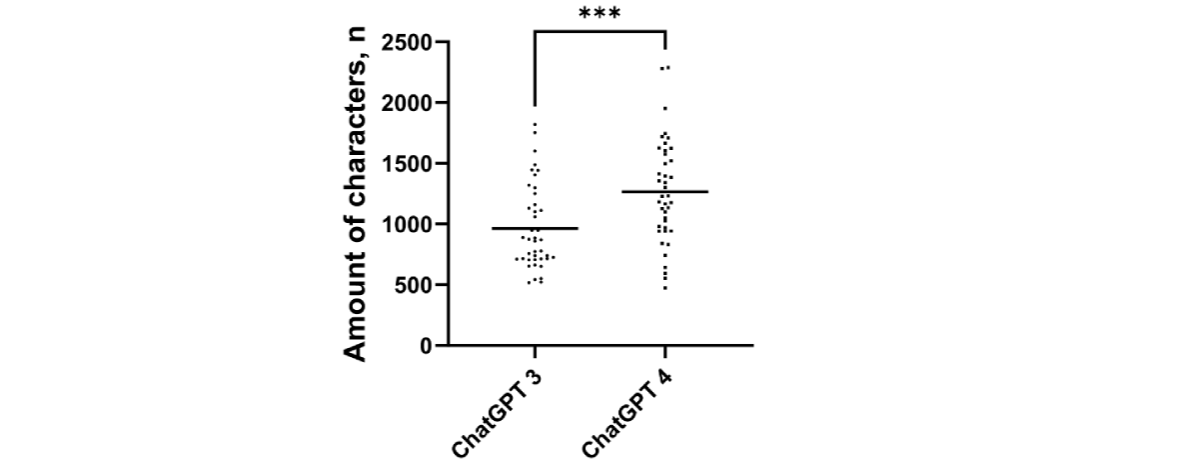
The number of characters used by ChatGPT 3 and ChatGPT 4. Data shown as a scatter dot blot with each point resembling an absolute value. Horizontal lines represent the mean. The Welch 2-tailed t test was used to compare the 2 groups. ***P<.001.

On the other hand, neither the conciseness (*P*=.06) nor the comprehensibility (*P*=.08) improved significantly (Figure 4), whereas the number of characters significantly increased by 52.5% (n= (1470-964)/964; *P*<.001; Figure 5) when using the most recent version of ChatGPT.

## Discussion

### Principal Findings

This pilot study assessed the performance of the ChatGPT LLM in answering clinical case–based questions in the field of ORL and compared it with certified ORL consultants. Overall, the ORL consultants significantly outperformed ChatGPT in medical adequacy, conciseness, comprehensibility, and coherence (Table 1 and Figure 2).

### Comparison to Prior Work

Medical adequacy should be considered the most critical parameter, as even minor inaccuracies can lead to misdiagnosis or misinterpretation, resulting in increased anxiety, incorrect conclusions, and inadequate therapy or nontherapy [17,18]. Despite being an open access service without specific medical training, ChatGPT achieved relatively high ratings for medical adequacy. However, it still lagged behind the ORL consultants. ChatGPT’s high-quality language output and coherent answers could potentially mislead users into believing they are receiving medically accurate information due to the halo effect [25]. This is concerning, especially since patients may struggle to interpret and apply the generated advice without physical examination, specialized tests, or clinical consultation. In this study, 10.6% (13/123) of ChatGPT’s responses were rated “poor” or “very poor” in the category “medical adequacy” by at least 1 rater. Contrarily, only 1.2% (3/246) of answers by ORL consultants in “medical adequacy” were rated in the worst categories. This emphasizes the significance of a potential hazard caused by inadequate answers provided by ChatGPT. For instance, ChatGPT did conclude allergic symptoms in response to a case evolving around a potentially life-threatening cutaneous abscess, which was adequately recognized by all ORL consultants.

Moreover, ChatGPT’s inability to recognize nonverbal cues or misunderstandings further highlights the limitations of LLMs in comparison to human physicians [26,27]. The high ratings for coherence and comprehensibility of ChatGPT’s responses emphasize its semantic output quality but do not guarantee medical accuracy. In this study, ORL consultants easily distinguished between LLM-generated and human answers, indicating that ChatGPT failed a simplified Turing test [28]. Although ORL consultants knew that 1 answer was generated by a machine, which represents a potential study bias, the high recognition rate is still relevant. The recognizability may be explained by an answering style consisting of long answers and a wording and semantic structure characteristic for ChatGPT (see Example S1 in Multimedia Appendix 1). Nevertheless, laypeople might be more susceptible to ChatGPT’s eloquence.

Despite ChatGPT’s inferior performance in all evaluated categories, the potential for future improvements cannot be ignored. LLM-based chatbots such as ChatGPT could revolutionize clinical care by increasing the availability of medical information, especially in low-resource settings. As new and improved LLMs are developed, their medical accuracy may improve, making them valuable augmentative tools for medical professionals. This could lead to more precise, time-efficient, and individualized medicine.

### Strengths and Limitations

However, the current limitations of LLMs, such as data protection and legal issues, must be addressed before they can be integrated into clinical practice [29-31]. This study design has certain limitations. First, the use of case-based questions does not properly reflect the style or quality of laypeople questions. Furthermore, considering the accuracy of identification of the source of the answers provided may influence the rating and limit the characterization as a “single blinded study.” Although the questions were specifically selected in concordance with equivalent cases in the ORL department, using text-based questions is an obvious limitation of this study design.

Furthermore, the evolution in the field of LLMs is progressing rapidly. Thus, all scientific data obtained in this field will ultimately only be able to depict a specific time point of LLMs evolving potential.

### Future Directions

While early LLMs were trained on small data sets of text and code, they often generated rather inaccurate answers. Yet, a significant increase in the size and complexity of LLM data sets resulted in improvements in the accuracy and reliability of LLM-generated medical answers [32]. To allow an insight into the current state of the art of LLMs, a comparison of findings obtained with the latest (fee-based) version of ChatGPT (ChatGPT 4) to ChatGPT 3 was added during the review process. As shown in Figure 4, the latest version showed only a slight improvement for medical adequacy (*P*=.03). Yet, ChatGPT 4 was rated with a better coherence of the answers provided when compared to ChatGPT 3 (*P*=.005). In contrast, the conciseness or comprehensibility did not improve significantly although the amount of characters increased by a highly significant 52.5% (n=(1470-964)/964; Figure 5). These findings are in concordance with recent published data highlighting that LLMs often generate inconcise answers due to the sheer amount of information provided [33]. Through years of training, clinicians also possess a large knowledge base concerning their specific field. Ultimately, soft skills such as the identification of nonverbal communication as personal experience cannot be replicated by an LLM. In clinical practice, therapeutic decisions are rarely based on the anamnesis alone. Instead, clinicians can gather a multidimensional view of the patients’ pathology combing findings of the anamnesis, examinations, and personal impressions. These are advantages an LLM currently simply cannot match. We believe that the rapid evolution of LLMs will soon provide better and more specialized advice for medical problems, making them more relevant as an augmentative option especially in areas with insufficient availability of medical care. Nevertheless, we are convinced that human consultation will remain the undisputed gold standard in medical care in the near future.

Therefore, this pilot study serves as a starting point for evaluating the performance of LLMs in the field of ORL. Further research should investigate the potential of LLMs on a larger scale and for different audiences, focusing on the development of specialized LLMs that could assist health care professionals without replacing their expertise.

## Appendix

Multimedia Appendix 1 Example question and individual consultant comparisons.

## Abbreviations

AI: artificial intelligence
LMM: large language model
ORL: otorhinolaryngology
USMLE: United States Medical Licensing Examination
