# Supplementary material for: ChatGPT Versus Consultants: Blinded Evaluation on Answering Otorhinolaryngology Case–Based Questions
Source: JMIR Med Educ. 2023 Dec 5;9:e49183. doi: 10.2196/49183 (PMC10731554; doi:10.2196/49183)
Supplement: Multimedia Appendix 1 [file mededu_v9i1e49183_app1.docx]

**Example S1**

**Example Question retrieved from Reineke U, Riemann R. Facharztprüfung Hals-Nasen-Ohrenheilkunde: 1000 kommentierte Prüfungsfragen: Thieme; 2007. ISBN: 9783131582317 and translated to English:**

“A patient with a painful swelling of the tip of the nose and upper lip comes to your consultation. Trauma is denied. What could the patient suffer from?”

**Example answer generated by ChatGPT (and translated to English):**

“Painful swelling of the tip of the nose and upper lip may indicate various diseases. One possible cause of these symptoms is an infection of the sinuses, especially the maxillary sinus. Inflammation of the maxillary sinus can affect the nose and lips, causing pain and swelling.

Another possible cause of these symptoms is an allergic reaction. Allergies can cause swelling of the face, including the nose and lips.

Other causes of pain and swelling in these areas may include infection with herpes viruses, a reaction to insect bites, or a skin rash.

Since trauma has been ruled out, further investigation by a physician should be performed to determine the exact cause of the discomfort and initiate appropriate treatment.”

**Example answer given by an ORL consultant:**

“Depending on the duration and the dynamics of the onset of symptoms, a nasal furuncle/carbuncle or erysipelas up to phlegmonous inflammation appears as the primary most likely differential diagnosis.

A tumorous formation in or on the nose should also be considered as a differential diagnosis, especially if the symptoms occur over a long period of time.”


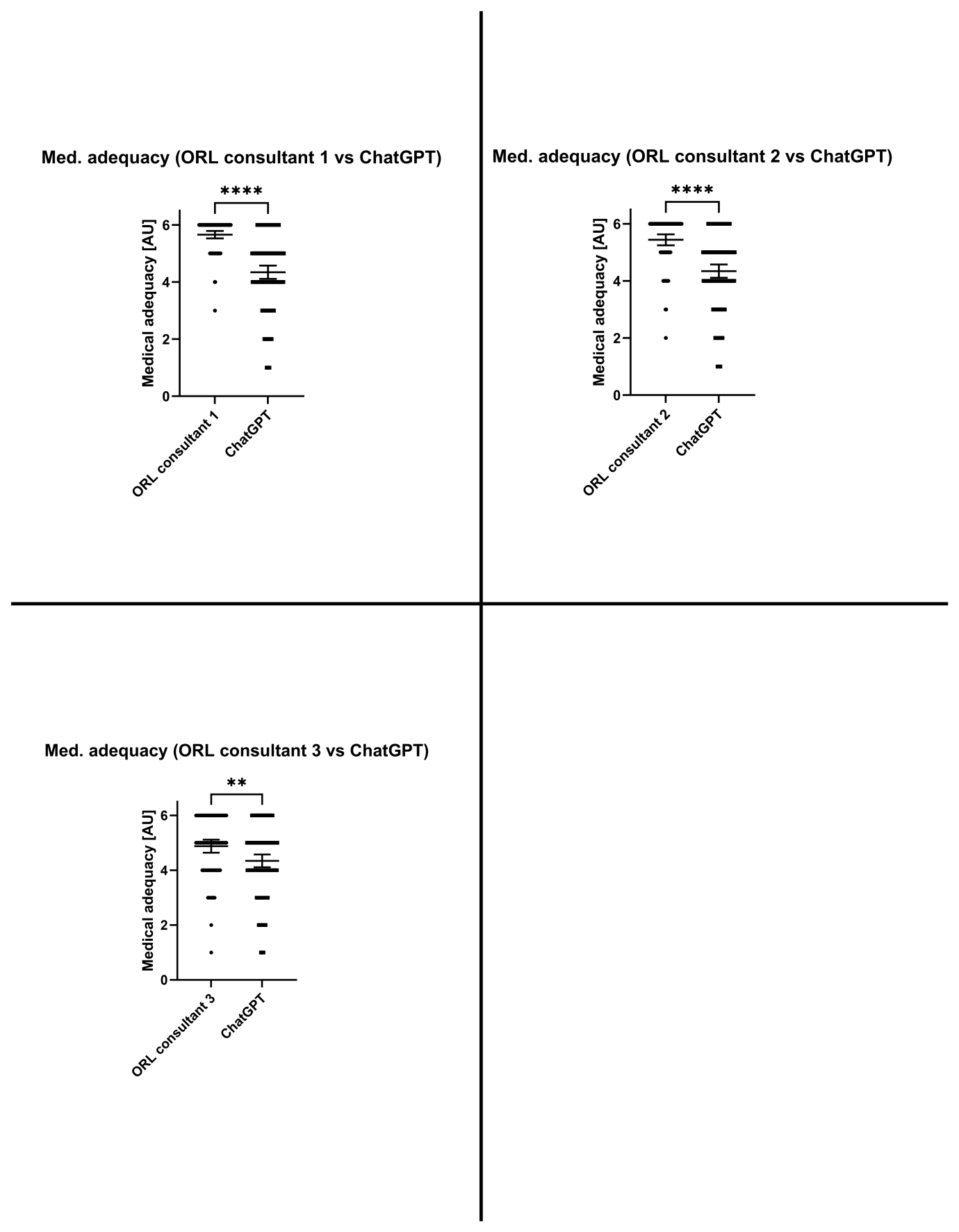


**Figure S1.** Data shown as a scatter dot blot with each point resembling an absolute value (Bar width resembling a high amount of individual values). Horizontal line = Mean ± 95% CI (****= p < .0001, **= p < .01).


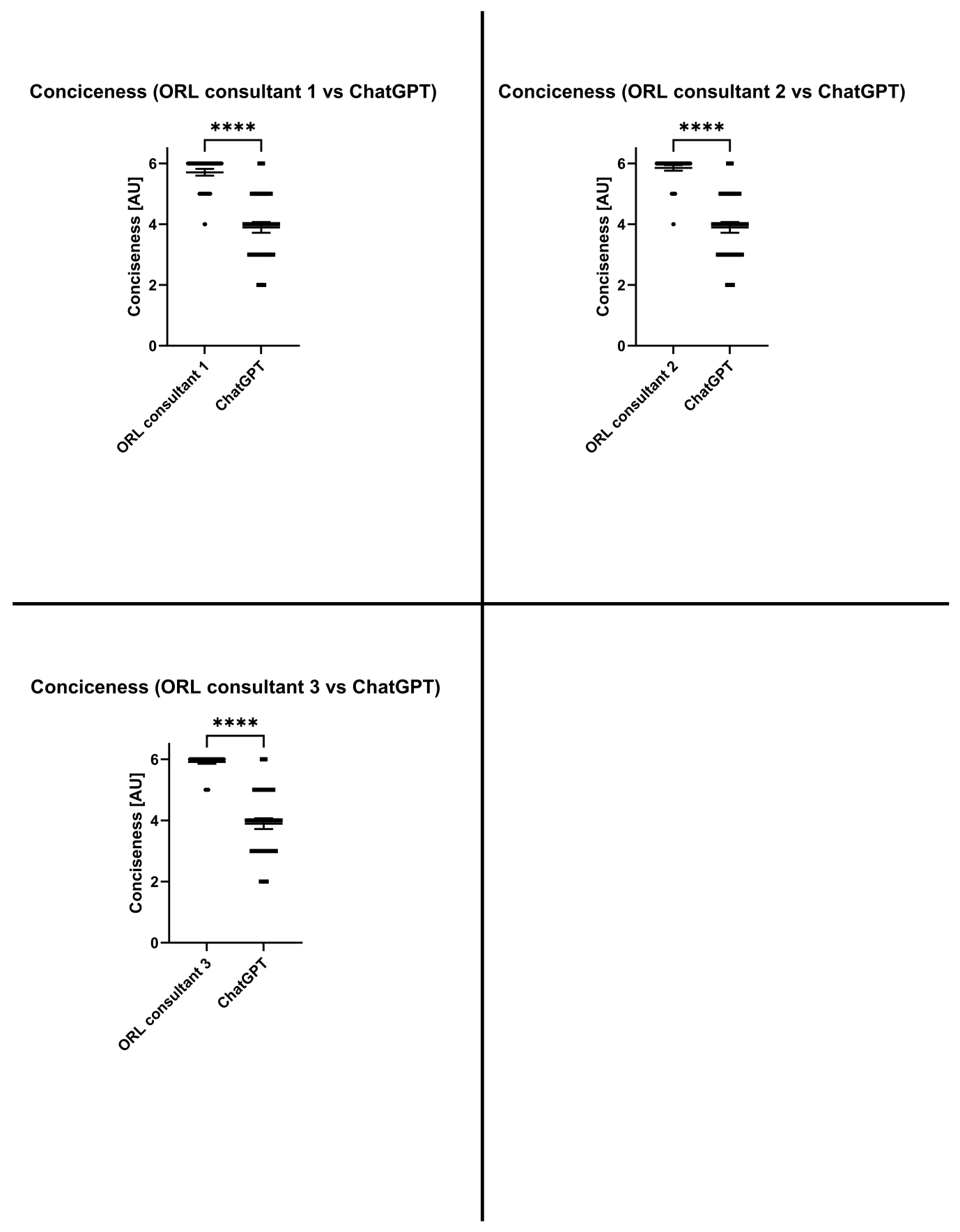


**Figure S2.** Data shown as a scatter dot blot with each point resembling an absolute value (Bar width resembling a high amount of individual values). Horizontal line = Mean ± 95% CI (****= p < .0001).


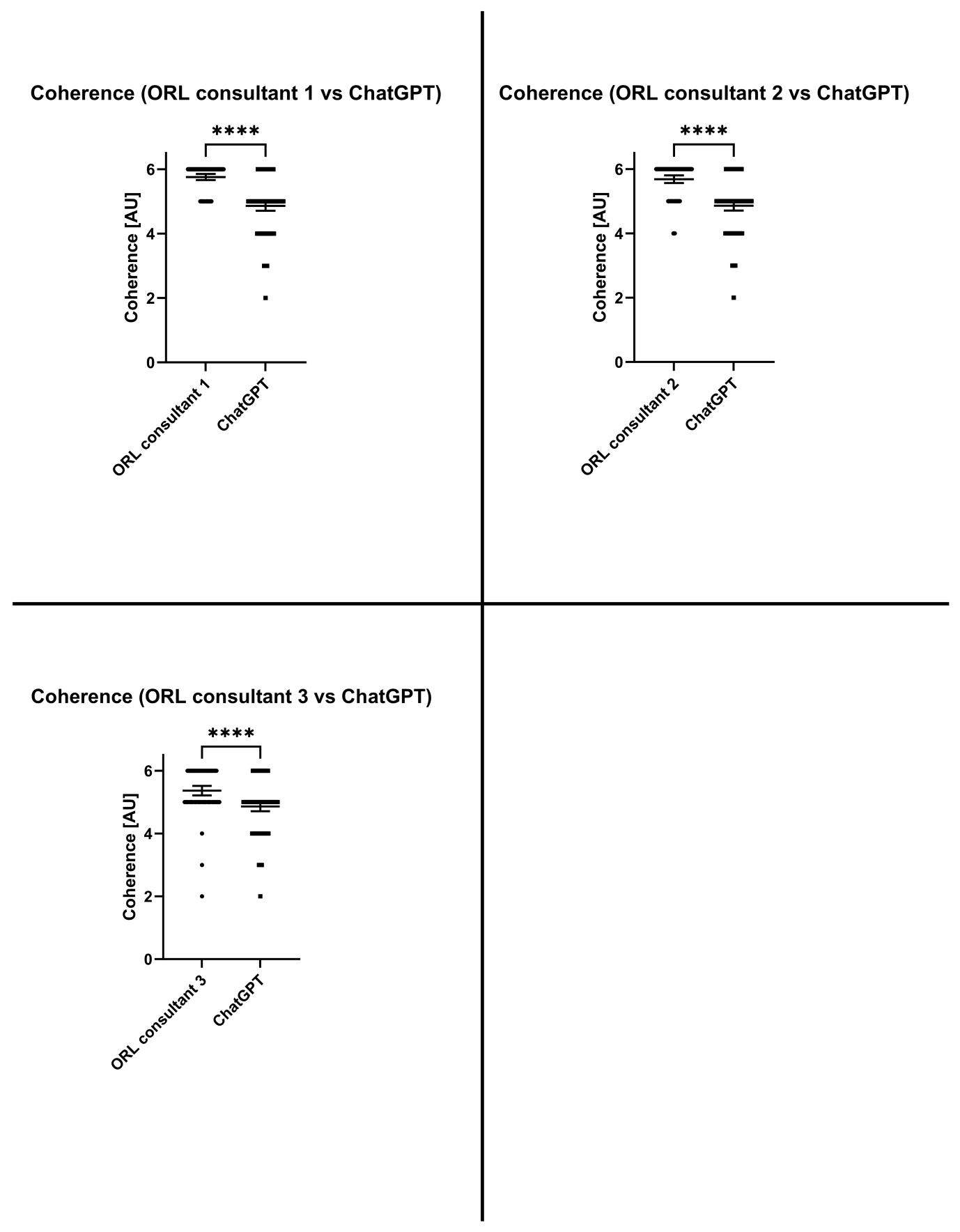


**Figure S3.** Data shown as a scatter dot blot with each point resembling an absolute value (Bar width resembling a high amount of individual values). Horizontal line = Mean ± 95% CI (****= p < .0001).


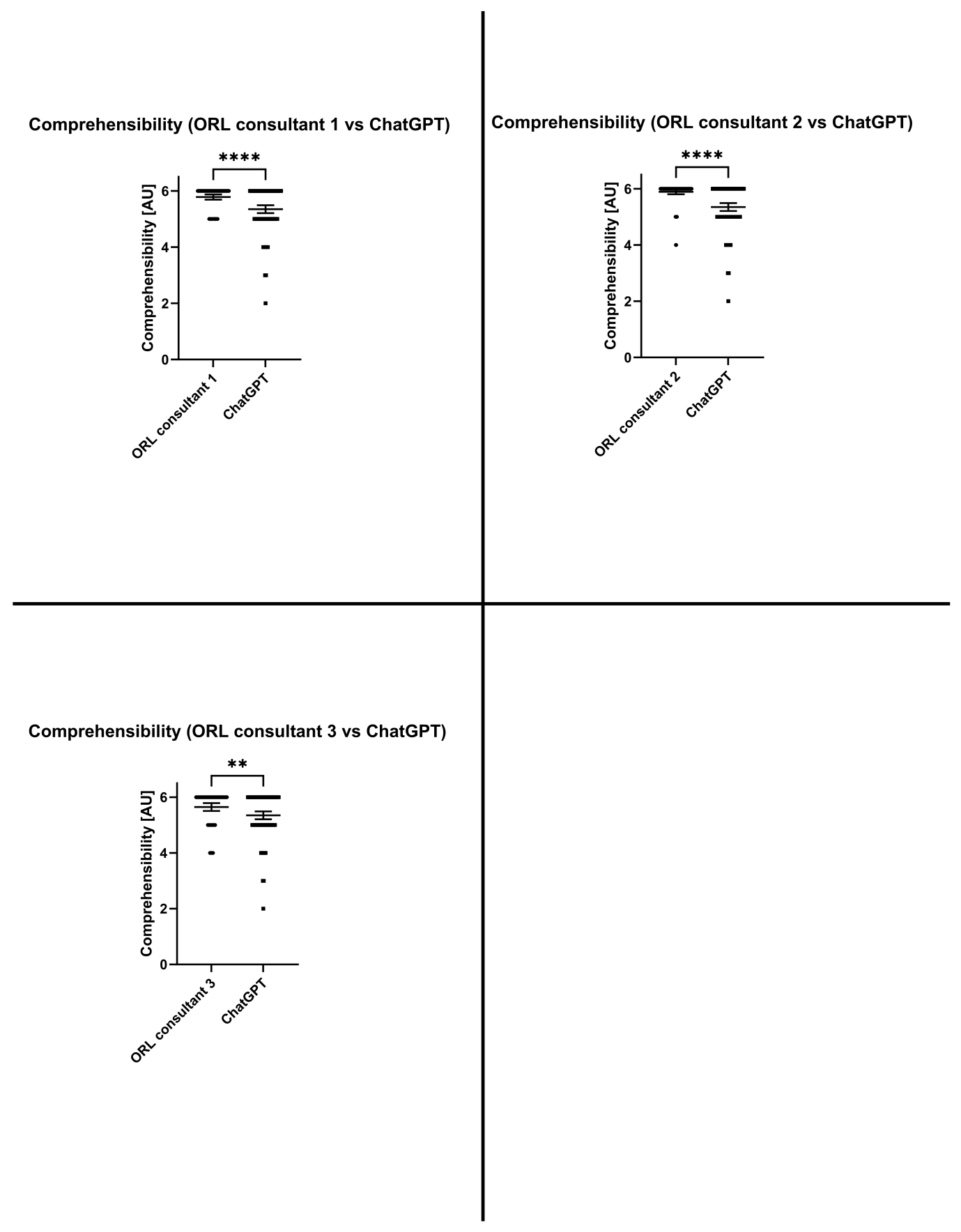


**Figure S4.** Data shown as a scatter dot blot with each point resembling an absolute value (Bar width resembling a high amount of individual values). Horizontal line = Mean ± 95% CI (****= p < .0001, **= p < .01).


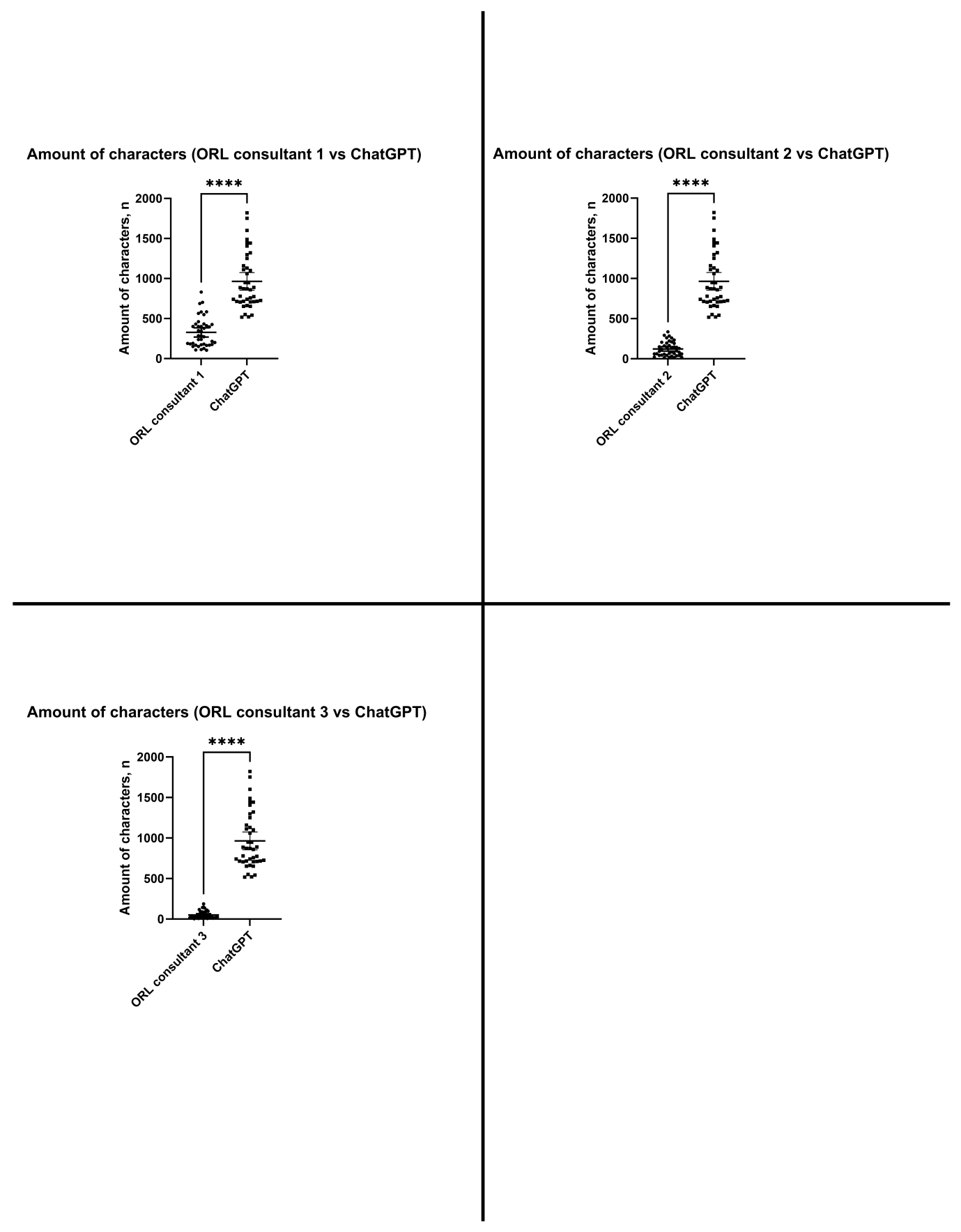


**Figure S5.** Data shown as a scatter dot blot with each point resembling an absolute value (Bar width resembling a high amount of individual values). Horizontal line = Mean ± 95% CI (****= p < .0001).
